# VEGF_121_, is predictor for survival in activated B-cell-like diffuse large B-cell lymphoma and is related to an immune response gene signature conserved in cancers

**DOI:** 10.18632/oncotarget.19385

**Published:** 2017-07-19

**Authors:** Julien Broséus, Samia Mourah, Gérard Ramstein, Sophie Bernard, Nicolas Mounier, Wendy Cuccuini, Philippe Gaulard, Christian Gisselbrecht, Josette Brière, Rémi Houlgatte, Catherine Thieblemont

**Affiliations:** ^1^ Inserm U954, Faculty of Medicine, University of Lorraine, Nancy, France; ^2^ University Hospital of Nancy, Hematology Laboratory, Nancy, France; ^3^ Paris Diderot University, Sorbonne Paris Cité, Paris, France; ^4^ APHP, Saint Louis University Hospital, Pharmacology-Biologic Laboratory, Paris, France; ^5^ Inserm UMRS 976, France; ^6^ LS2N - DUKe, University of Nantes, Nantes, France; ^7^ APHP, Saint-Louis University Hospital, Hemato-Oncology, Paris, France; ^8^ University Hospital of L’archet, Nice, France; ^9^ APHP, Saint-Louis University Hospital, Hematology Laboratory, Paris, France; ^10^ Department of Pathology, APHP, Henri Mondor University Hospital, Creteil, France; ^11^ Inserm U955, University Paris-Est, Créteil, France; ^12^ Lymphoma Study Association, Pierre-Bénite, France; ^13^ Department of Pathology, APHP, Saint-Louis University Hospital, Paris, France; ^14^ University Hospital of Nancy, DRCI, Nancy, France

**Keywords:** ABC, like DLBCL, angiogenesis, immune response, cancer

## Abstract

Tumor microenvironment including endothelial and immune cells plays a crucial role in tumor progression and has been shown to dramatically influence cancer survival. In this study, we investigated the clinical relevance of the gene expression of key mediators of angiogenesis, VEGF isoforms 121, 165, and 189, and their receptors (VEGFR-1 and R-2) in a cohort of patients (*n* = 37) with relapsed/refractory diffuse large B-cell lymphoma (DLBCL) from the Collaborative Trial in Relapsed Aggressive Lymphoma (CORAL). In patients with ABC-like DLBCL, but not in patients with GCB-like DLBCL, low *VEGF*_*121*_ expression was associated with a significantly better survival than in those with high *VEGF*_*121*_ level: 4-year overall survival at 100% vs 36% (*p* = .011), respectively. A specific gene signature including 57 genes was correlated to *VEGF*_*121*_ expression level and was analyzed using a discovery process in 1,842 GSE datasets of public microarray studies. This gene signature was significantly expressed in other cancer datasets and was associated with immune response. In conclusion, low *VEGF*_*121*_ expression level was significantly associated with a good prognosis in relapsed/refractory ABC-like DLBCL, and with a well-conserved gene-expression profiling signature related to immune response. These findings pave the way for rationalization of drugs targeting immune response in refractory/relapsed ABC-like DLBCL.

## INTRODUCTION

Tumor microenvironment plays a major role in tumor growth, with key players including immune cells, stromal cells, extracellular matrix and angiogenesis [1]. Angiogenesis is precisely regulated by genes encoding for the vascular endothelial growth factor (VEGF) and its receptors (VEGFR). *VEGF* (referred to also as VEGF-A) belongs to a gene family that includes placenta growth factor (*PlGF*), *VEGF-B*, *VEGF-C*, and *VEGF-D* [2]. VEGF has five main isoforms produced by alternative splicing of a gene located on 6p21.3: *VEGF*_*121*_, *VEGF*_*165*_, *VEGF*_*189*_, *VEGF*_*145*_, and *VEGF*_*206*_, which differ in their bioavailability [3]. *VEGF* mRNA is expressed in the vast majority of human tumors, including lung, breast, gastrointestinal tract, kidney, bladder, ovary, and endometrium carcinoma and several intracranial tumors including glioblastoma (see [3] for review). In the last 10 years, the clinical impact of *VEGF* expression has been a breakthrough, with an important link between tumor angiogenesis and survival, and the demonstration of a clinical benefit in inhibiting VEGF, increasing survival in patients with advanced malignancies.

In lymphoma, *VEGF* expression is frequently increased, and predicts a poor response to treatment [4-6]. Different analytic approaches by gene-expression profiling (GEP) identified distinct biologic attributes of diffuse large B-cell lymphoma (DLBCL) tumors that are associated with survival. The first GEP studies identified two biologically and clinically distinct molecular subtypes of DLBCL [7, 8]. The germinal-center B-cell-like DLBCL (GCB-like DLBCL) arises from normal germinal-center B-cells, whereas activated B-cell-like DLBCL (ABC-like DLBCL) arises from a post-germinal-center B cell that is blocked during plasmacytic differentiation. These two cell-of-origin (COO) subtypes have different oncogenic mechanisms and are responding differently to treatment [9, 10].

In the context of relapse, several adverse risk factors have been identified, such as International Prognosis Index (IPI), prior rituximab treatment, *c-MYC* gene rearrangment, COO subtype, and delay of relapse [10-12]. In addition, whole-exome sequencing and copy number variations (CNV) analysis by SNP array identified frequent abnormalities, some of which holding a prognostic value. Abnormalities affect genes related to cell cycle and apoptosis (*TP53, CDKN2A, MYC, DIABLO, PTMS, CK2B, XPO1, RB1, FAT2, ATM, CCND3*), chromatin modifications (*KMT2D*, *EZH2, CREBBP, HIST1 H1T/H2BC/H2AK, AIRN, SMARCA4, TBL1XR1, MLL3*), cell proliferation (*HES1*, *DVL3*,*TMSB4X*, *HYAL2*), B-cell development and immune response (*CD58*, *B2M, PRDM1, REL, GNA13, IRF4, BCL2, LGALS9C, CIITA, POU2AF1, IGLL5*), BCR signalling (*IBTK*, CD79B, *FOXO1*, *PTPN6*), NFKB pathway (*MYD88, CARD11, PIM1, TNFAIP3, NFKBIA, NFKBIE, NFKBIZ*), MAPK pathway (*DUSP2*), JAK-STAT pathway (*STAT6, SOCS1*), insulin secretion pathway (*PCLO*) and tryptophan degradation pathway (*IDO1*, *TDO2*) [13-19].

A second analytic approach identified the prognostic impact of the tumor microenvironment [8, 20, 21]. Two gene-expression signatures, stromal-1 and stromal-2, reflecting the character of non-malignant cells in DLBCL, were identified as significant prognostic factors. The stromal-1 signature, reflecting extracellular matrix, fibrotic reaction, and histiocyte and myeloid cells infiltration, was associated with a favourable prognosis. The stromal-2 signature, reflecting blood-vessel density and angiogenic activity, was associated with unfavourable prognosis in patients treated by the standard R-CHOP (Rituximab, Cyclophosphamide, Adriamycin, Vincristine and Prednisone) regimen [20].

Our goal was to evaluate the clinical impact of the expression of *VEGF* isoforms, *VEGF*_*121*_, *VEGF*_*165*_, *VEGF*_*189*_, and their receptors *VEGFR-1* and *R-2* in a cohort of patients with relapsed/refractory DLBCL prospectively treated in the international multicentre trial CORAL (Collaborative Trial in Relapsed Aggressive Lymphoma) [11]. We secondary aimed at exploring the biological significance of the differential expression of *VEGF*_*121*_, the only isoform with a clinical impact in our series, by performing a GEP analysis. We identified a specific gene signature, validated this gene signature in all public cancer datasets available and characterized its function.

## RESULTS

### Low level of soluble VEGF_121_ mRNA is significantly associated with a better prognosis in ABC-like DLBCL

The expression levels of the 5 transcripts *VEGF*_*121*_, *VEGF*_*165*,_ *VEGF*_*189*_, *VEGFR-1* and *VEGFR-2* are described in the Table 1. In the whole cohort, *VEGF*_*121*_ expression below the median level was associated with a better outcome, with a 4-year progression-free survival (PFS) at 63% *vs* 33% (*p* = .0533) and a 4-year overall survival (OS) at 79% *vs* 37% (*p* = .0321), respectively. *VEGF*_*165*_, *VEGF*_*189*_, and *VEGF-R1*, *-R2* transcript levels did not have any significant impact (Table 2).

**Table 1:** Expression of *VEGF-A* isoforms (121, 165, 189) and receptors (R1 and R2) in patients with relapsed/refractory DLBCL.

	Median	Mean	range	SD	min	max
**VEGF121** All DLBCL	0.6248	1.2474.	9.6844	1.9591	0.0565	9.7409
GCB-like DLBCL	0.5560	1.4466	9.6844	2.5584	0.0565	9.7409
ABC-like DLBCL	0.7184	1.0302	4.5210	1.1208	0.0613	4.5824
**VEGF165** All DLBCL	0.0801	0.1423	1.2156	0.2206	0.0072	1.2228
GCB-like DLBCL	0.0691	0.1617	1.1978	0.2834	0.0250	1.2228
ABC-like DLBCL	0.0858	0.1229	0.5869	0.1381	0.0072	0.5941
**VEGF189** All DLBCL	0.0381	0.0628	0.5237	0.0912	0.0099	0.5337
GCB-like DLBCL	0.0293	0.0624	0.5194	0.1186	0.0142	0.5337
ABC-like DLBCL	0.0491	0.0631	0.2277	0.0553	0.0099	0.2376
**VEGFR-1** All DLBCL	0.3047	0.48029	2.3876	0.5609	0.0683	2.4560
GCB-like DLBCL	0.3376	0.5738	2.3876	0.6572	0.0683	2.4560
ABC-like DLBCL	0.2604	0.3867	1.6154	0.4441	0.0688	1.6842
**VEGFR-2** All DLBCL	0.1445	0.2249	1.7402	0.0941	0.0163	1.7566
GCB-like DLBCL	0.1914	0.2356	0.7779	0.1930	0.0163	1.7566
ABC-like DLBCL	0.0986	0.2143	1.7402	0.3954	0.0370	0.8149

Results are shown in the all group of patients and regarding the subtypes ABC-like DLBCL (*n* = 18) and GCB-like DLBCL (*n* = 19). SD: Standard Deviation.

**Table 2:** Impact of cell of origin classification, gene-expression profiling indexes and level of angiogenic biomarkers.

Number of patients (n=37)	n	(%)	4-Year PFS	p	4-Year OS	p
**Cell of origin**						
GC	19	(51)	58	.3551	57	.6412
ABC	18	(49)	39		61	
**TGS** = (-0.32 x *LMO2*) + (-0.29 x *TNFRSF9*)						
Low risk (TGS < -1.60)	0	(0)				
Int risk (- 0.91 > TGS > -1.60)	21	(32)	67	.3017	44	.7916
High risk (TGS > -0.91)	25	(68)	40		55	
**TGS-IPI** =(0.93 x TGS)+(0.6xIPI)+4						
Low risk (TGS-IPI < 3.47)	0	(0)	-	-	-	-
Int risk (4.51 > TGS-IPI > 3.47)	0	(0)				
High risk (TGS-IPI > 4.51)	37	(37)				
**VEGF121**						
Low level	19	(51)	63	.0533	79	.0321
High level	18	(49)	33		37	
**VEGF165**						
Low level	18	(49)	61	.1094	77	.0673
High level	19	(51)	37		47	
**VEGF189**						
Low level	19	(51)	53	.5535	63	.8295
High level	18	(49)	44		56	
**VEGFR-1**						
Low level	19	(51)	53	.7055	63	.8851
High level	18	(49)	44		55	
**VEGFR-2**						
Low level	18	(49)	50	.9458	61	.9200
High level	19	(51)	47		57	

GC: Germinal Center B-cell like; ABC: Activated B-cell like; TGS: Two-Gene Score; IPI: International Prognosis Factor; PFS: Progression-Free Survival; OS: Overall Survival; VEGF: Vascular Endothelial Growth Factor.

**Figure 1:**
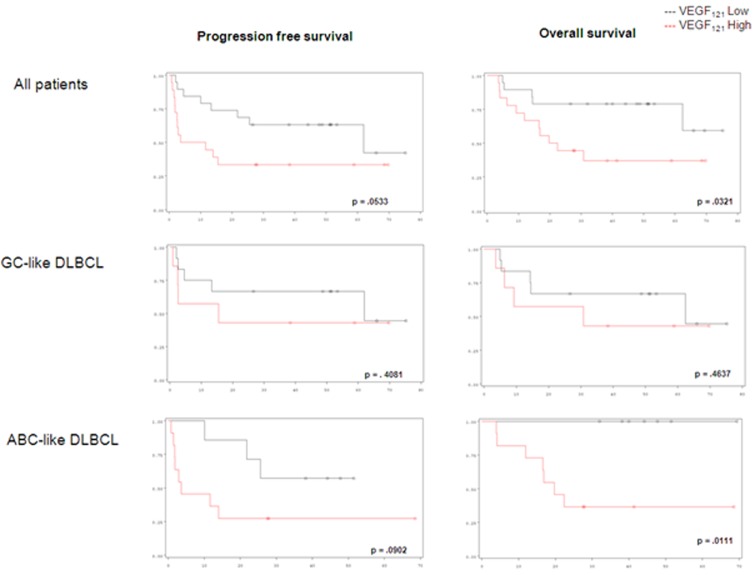
Progression-free survival and overall survival in DLBCL patients considering VEGF_121_ expression level

Eighteen patients were predicted as ABC-like DLBCL and 19 as GCB-like DLBCL. In patients with ABC-like DLBCL, low *VEGF*_*121*_ level was associated with a significantly better survival than in those with high *VEGF*_*121*_ level: 4 year-PFS at 57% *vs* 27%, *p* = .0533 and 4-year OS at 100% *vs* 36% (*p* = .0111). The differences in outcome according to *VEGF* isoforms were not significant among patients with GCB-like DLBCL (Figure 1).

The prognostic value of *VEGF*_*121*_ expression level was analysed regarding the clinical and biological characteristics of the cohort of patients by multivariate analysis. None of the GEP scores ie COO and TGS (Two-Gene Score) influenced the prognosis in this subset of population (Table 2). The type of induction treatment did not influence the outcome, and the prognostic value of *VEGF*_*121*_ expression level was not observed whatever the type of treatment. Beside *VEGF*_*121*_ expression, two other biological parameters influenced the overall survival: (i) the presence of *MYC* rearrangement detected by FISH (Fluoresence In Situ Hybridization) analysis (*p* = .0540) as already demonstrated [12] with limited statistical significance because of the low number of occurrence, and (ii) the functional status of p53 (*p* = .0360).

### Soluble VEGF_121_ transcript level is specifically associated to a specific gene signature

**Figure 2:**
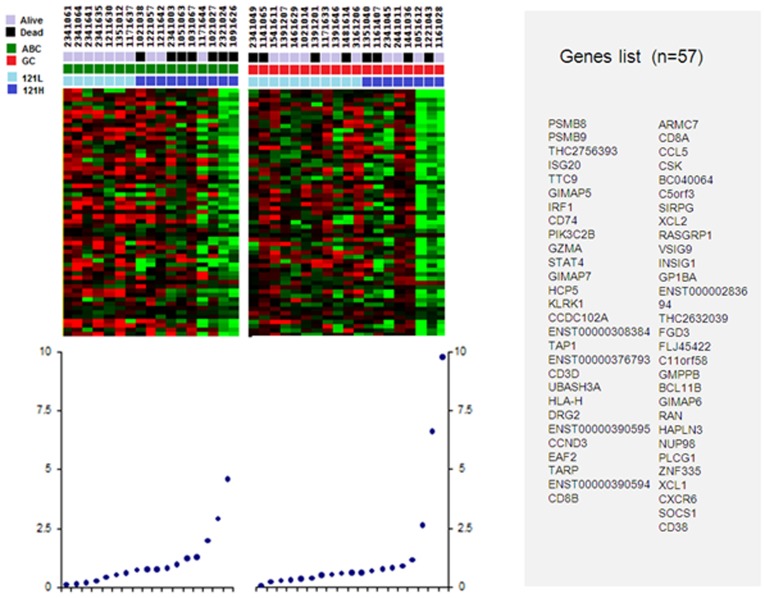
Genes set correlating to low and high levels of VEGF_121_ in the ABC-like and GCB-like DLBCL Light blue squares define low level of *VEGF*_*121*_ (121L); dark blue squares, high level of *VEGF*_*121*_ (121H). Red squares define ABC-like DLBCL samples; green square GCB-like DLBCL samples. Black squares define dead patients, and grey squares define alive patients.

Gene signature associated to soluble *VEGF*_*121*_ differential expression grouped 57 genes listed in Figure 2. This signature was associated with higher level of *VEGF*_*121*_ in both ABC-like and GCB-like samples, but with worse outcome only in ABC-like samples. All these genes were under-expressed in high *VEGF*_*121*_-expressing samples. Functional annotations of these 57 genes showed that these genes are involved in immune response and T-cell activation (Table 3).

**Table 3:** Functional annotations of the 57 genes of the gene signature associated to soluble VEGF_121_ differential expression.

#GO	GO name	Total genes	Changed genes	Enrichment	Log10(p)	FDR
GO:0002376	Immune system process	921	22	5.440415	11.383116	0.000000
GO:0042110	T cell activation	209	12	13.076874	10.221357	0.000000
GO:0046649	Lymphocyte activation	279	13	10.612266	-9.941860	0.000000
GO:0001775	Cell activation	452	15	7.558260	-9.424698	0.000000
GO:0045321	Leukocyte activation	318	13	9.310762	-9.238159	0.000000
GO:0006955	Immune response	567	16	6.426965	-9.044632	0.000000
GO:0042287	MHC protein binding	13	5	87.598291	-8.788562	0.000000
GO:0002682	Regulation of immune system process	461	14	6.916655	-8.277422	0.000000
GO:0048583	Regulation of response to stimulus	516	14	6.179414	-7.653025	0.000000
GO:0042288	MHC class I protein binding	10	4	91.102222	-7.175224	0.000000
GO:0050776	Regulation of immune response	282	10	8.076438	-6.539483	0.000000
GO:0023052	signaling	2301	26	2.573509	-6.512071	0.000000
GO:0002684	Positive regulation of immune system process	315	10	7.230335	-6.096855	0.000000
GO:0051249	Regulation of lymphocyte activation	181	8	10.066544	-6.000050	0.000000
GO:0030217	T cell differentiation	82	6	16.665041	-5.858916	0.000000
GO:0002694	Regulation of leukocyte activation	194	8	9.391982	-5.772747	0.000000
GO:0048518	Positive regulation of biological process	1680	21	2.846944	-5.707682	0.000000
GO:0019882	antigen processing and presentation	49	5	23.240363	-5.668759	0.000000
GO:0051251	Positive regulation of lymphocyte activation	141	7	11.307013	-5.627840	0.000000
GO:0050865	Regulation of cell activation	204	8	8.931590	-5.609057	0.000000
GO:0050778	Positive regulation of immune response	205	8	8.888022	-5.593179	0.000000
GO:0002696	Positive regulation of leukocyte activation	146	7	10.919787	-5.526712	0.000000
GO:0048584	Positive regulation of response to stimulus	286	9	7.167133	-5.479161	0.000000
GO:0050863	Regulation of T cell activation	149	7	10.699925	-5.467838	0.000000
GO:0050867	Positive regulation of cell activation	151	7	10.558205	-5.429306	0.000000
GO:0002521	Leukocyte differentiation	152	7	10.488743	-5.410249	0.000000
GO:0002429	Immune response-activating cell surface receptor signaling pathway	99	6	13.803367	-5.377925	0.000000
GO:0002768	Immune response-regulating cell surface receptor signaling pathway	102	6	13.397386	-5.302391	0.000000
GO:0023033	Signaling pathway	1801	21	2.655673	-5.208275	0.000000
GO:0050896	Response to stimulus	2482	25	2.294073	-5.207908	0.000000
GO:0030098	Lymphocyte differentiation	109	6	12.537003	-5.135135	0.000000

MHC: Major Histocompatibility Complex; FDR: False Discovery Rate; GO: Gene Ontology.

### The specific gene signature is conserved in public cancer datasets

**Figure 3:**
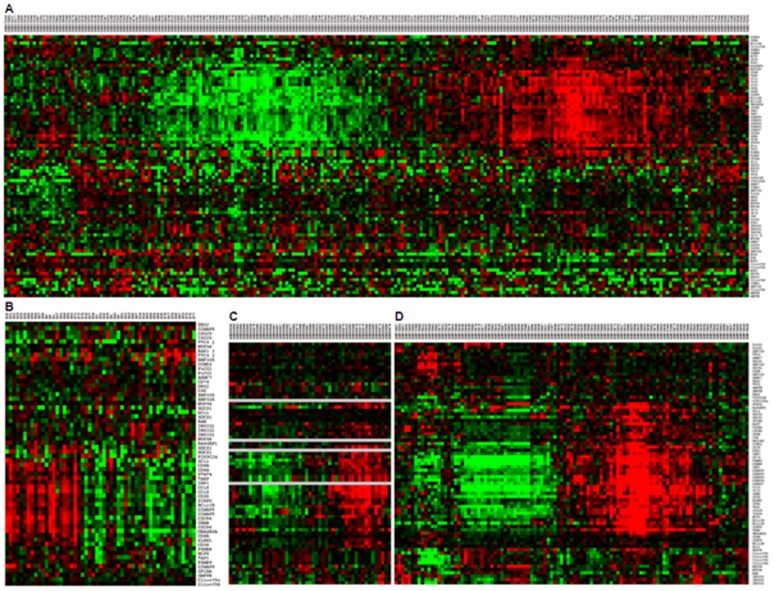
Conservation of the signature in public datasets Gene expression signature associated to soluble *VEGF*_*121*_ expression were found in public data sets. The signature was found in two DLBCL studies (GSE10846 [20] Figure 3A; E-TABM-346 [23] Figure 3B) and two positive studies found in GEO database by the previous strategy (Breast cancer GSE1561 [24] Figure 3C; Adult Male Germ Cell Tumors GSE3218, [25] Figure 3D).

From the GEO (Gene Expression Omnibus) database, we considered human microarray platforms having a sufficient coverage of our gene set: GPL96 (84%), GPL570 (94%) and GPL571 (84%), and we retained 1,842 GSE (GEO Series) from these platforms. In these series, we searched for pairs of samples with the same trend of expression for our 57 genes, according to an already published method that identified from several different studies a common gene signature associated with tolerance to renal allograft [22]. For each pair of samples, we computed a p-value corresponding to the probability to observe the same trend by chance. The resulting pair of samples was ranked according to its p-value. We selected the 250 best pairs, with a threshold corresponding to a proportion *p* of positive expression changes greater or equal to 90%, a p-value less than 1.9 x 10^-8^ and an adjusted p-value for multiple comparisons less than 3.0 x 10^-5^ (using Holm method). We performed a text mining processing of the annotations of our GSE and compared the term occurrences between the series related to our selection and the remaining series. Using a Fisher test, we could notably associate to our selection the following significant terms: *tumor*, *carcinoma*, *immunity*, *lymphocyte*. Figure 3 shows the existence of this signature in two DLBCL studies (GSE10846 [20], Figure 3A; E-TABM-346 [23], Figure 3B) and two solid cancers studies (Breast cancer GSE1561 [24], Figure 3C; Adult Male Germ Cell Tumors GSE3218 [25], Figure 3D). This shows that more than half the genes are highly correlated in these studies and constitute a robust gene signature.

### The gene set function is related to immune response

**Figure 4:**
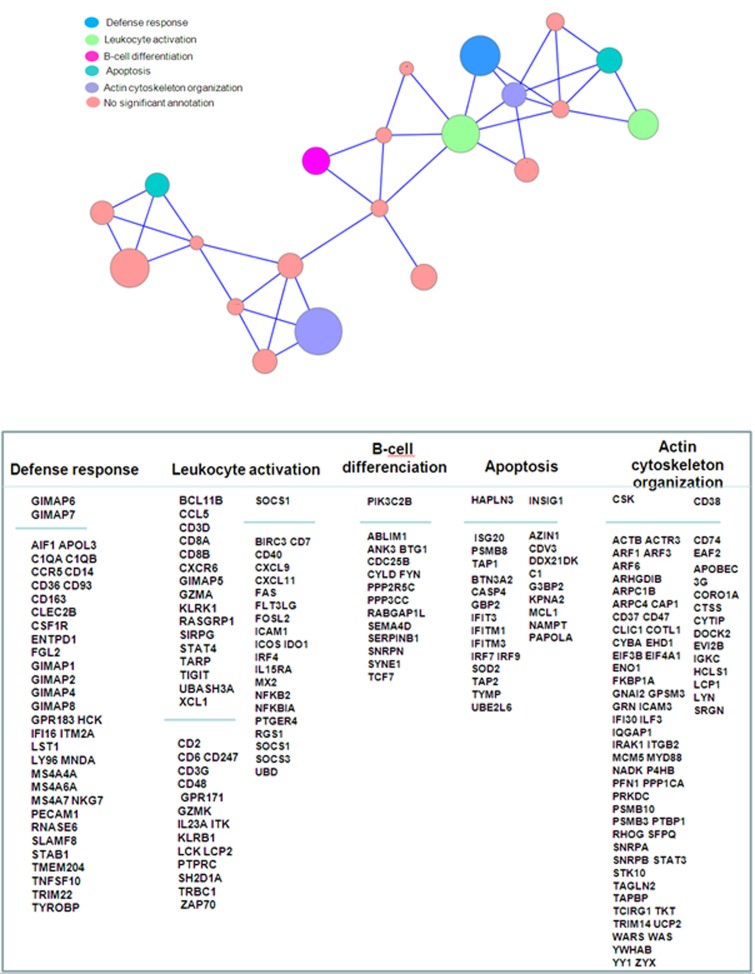
Summary graph of co-expressed genes Nodes represents clusters of genes frequently co-expressed in GEO studies. Node sizes are proportional to the number of genes aggregated in each node (mean cluster size=34 genes; min=5 genes; max=90 genes). Genes were aggregated as long as they are maximally connected to the other genes contained in the joined clusters, as explained in material & methods section. An edge links two clusters if their inter-cluster connectivity is greater or equal to 0.2. Gene names displayed in the upper part of the columns corresponds to the genes in our primary list; gene names in lower part correspond to a focus on the most conserved neighbors. This selection of neighbors was based on the median number of GEO series in which they were significantly co-expressed with a gene of our list, according to the MADCOW tool (296 neighbors are shown, corresponding to a median number of GEO series ranging from 34 to 138, with a mean of 81). Annotation indicates the most significant clusters. They denote over-representations of GO categories in a cluster compared to the whole gene set (*p*-values obtained by Fisher test; significance level of 0.05).

Using the MADCOW query engine [26], we searched for genes repetitively correlated to our gene list. Only 48 genes gave positive results and provided a set of 2,812 neighbors. We reduced the size of this set by discarding isolated neighbors. For this, we selected only genes that were the neighbor of at least 3 genes of our gene list. This filter retained 872 neighbors. We performed the hierarchical clustering of the genes and removed clusters containing less than 3 genes. Figure 4 represents the corresponding summary graph, consisting of 21 clusters related to a set of 719 genes. The functions of these gene clusters were related to tumor microenvironment including 5 majors clusters related to (i) defense response, (ii) leukocyte activation, (iii) B-cell differentiation, (iv) apoptosis and (v) actin cytoskeleton organization. The other clusters were too small to give significant functional annotation.

## DISCUSSION

Only a few studies have reported on the role of VEGF in lymphoma. In our study, the role of VEGF was analyzed in patients with relapsed/refractory DLBCL after R-CHOP. For these patients, prognosis is poor and new therapeutic strategies are urgently needed [27]. Here we demonstrate that the transcript level of the soluble isoform of *VEGF*, *VEGF*_*121*_, has a major impact on the prognosis of ABC-like DLBCL and is associated to a gene signature conserved in all cancer subtypes with a function related to the immune response.

Our results are in keeping with two recent studies on the prognostic impact of VEGF expression in DLBCL. In a meta-analysis of 8 studies (670 patients), positive VEGF protein expression in blood circulating lymphocytes and lymph nodes correlated with shorter survival in newly diagnosed DLBCL [28]. In another recent study performed on 149 newly diagnosed DLBCL, high serum VEGF level was associated with poorer prognosis [29]. Yet, our study is the first conducted on the different VEGF isoforms and receptors, on native tumor, in the context of relapsed DLBCL.

In our study, the prognostic impact of *VEGF*_*121*_ expression level was significant in ABC-like subtype and not in GCB-like subtype. These two DLBCL subtypes are well-known to be two distinct diseases with different oncogenic mechanisms [30]. The ABC subtype has gene-expression characteristics of normal B cells that were activated by cross-linking the B-cell receptor (BCR) [7]. The chronic active BCR signaling is a critical step in the pathogenesis of the ABC subtype [31, 32] and is associated to a constitutive NF-kB activation with the genetic alterations of 3 main actors: CARD11, BCL10, and MALT1. It has been shown that BCR signaling could directly interact with the microenvironment by decreasing the expression of CXCR4 and CD62L, two major players of nodal and marrow stroma in chronic lymphocytic leukemia [33].

Tumor microenvironment is a main battleground during the neoplastic process such as lymphoma, fostering proliferation and survival of tumor cells. This microenvironment is composed of immune cells, tumor cells, stromal cells and extracellular matrix. Angiogenesis is a key player in this ground, and is stimulated by angiogenic factors such as VEGF, produced by the tumoral cells. In the other hand, the tumoral cells are crosstalking to the immune system to propagate conditions that favour tumor immune tolerance and survival. We report here that the link between VEGF and immune response is conserved among several types of cancer, based on a specific gene signature. The main genes involved in this signature are grouped in clusters related to “immune defense”, “leucocyte activation” and “B-cell activation”. In the “immune defense” cluster, the discriminated genes were *GIMAP1*, *GIMAP2*, *GIMAP4*, *GIMAP6*, *GIMAP7*, *GIMAP8*, belonging the Gimap gene family shown to be integral to T-cell survival and development [34], *AIF1* (allograft inflammatory factor 1), associated with the inflammation and activated macrophages [35], and the C1q complement subunits C1QA and C1QB. The infiltrating macrophages may transmit trophic signals to the tumor, suppress antitumor immune responses, or both [36]. We identified 2 clusters related to “leucocyte activation”, one including discriminating genes such as *BCL11B*, *CCL5*, *CD3D*, *CD8A*, *CD8B*, *CXCR6*, and the other one including discriminating genes such as *SOCS1*, *BIRC3*, *CD7*, *CD40*, *CXCL9*, *CXCL11*, *FAS*, *FLT3LG*, *FOSL2*, *ICAM1*, *ICOS*, *IDO1*, *IRF4*, *IL15RA*, *MX2, NFKB2*, *NFKBIA*, *PTGER4*, *RGS1*. In the first cluster, *B-cell leukemia/lymphoma 11B* (*BCL11B*) is a member of the BCL family and plays a crucial role in the development, proliferation, differentiation and subsequent survival of T-cells. *BCL11B* alterations are related to malignant T-cell transformation that occurs in hematological malignancies, regulating the apoptotic process and cell proliferation [37]. SOCS1 (Suppressor Of Cytokine Signaling 1) in the second cluster, a member of the STAT-induced STAT Inhibitor (SSI), acts as cytokine-inducible negative regulator of cytokine signaling, downstream of cytokine receptors, and takes part in a negative feedback loop to attenuate cytokine signaling. In the “B-cell activation” cluster, *PIK3C2B* gene encodes for a phosphoinositide 3-kinase (PI3K) that plays a role in cell survival, proliferation, migration, and oncogenic transformation. Remarkably, the other clusters closely related to these 3 immune responses clusters, were linked to the organization of the cytoskeleton and the microenvironment.

Prognosis of patients with relapsed/refractory DLBCL is poor [11] and is strongly influenced by *MYC* rearrangements [12]. Response to standard regimen for relapse: R-ICE (Rituximab, Ifosfamide, Carboplatine, Etoposide) or R-DHAP (Rituximab, Dexamethasone, Cytarabine, Cisplatine) is different regarding the COO [10]. Our findings have major implications for new therapeutic strategies. Various VEGF signal inhibitors, including anti-VEGF neutralizing antibodies and VEGFR kinase/multi-kinase inhibitors, have been successfully developed and are now widely used in the clinic, particularly for colorectal cancer, lung cancer, breast cancer, glioblastoma, liver cancer and renal cell carcinoma treatment [38-40]. In preclinical studies performed on lymphoma xenografts, administration of an anti-VEGF antibody led to tumor regression, showing a synergistic antitumor effect with rituximab [41]. Recently, the efficacy and toxicity of rituximab-bevacizumab association versus single-agent rituximab was compared in patients with previously treated follicular lymphoma. The addition of bevacizumab to rituximab significantly improved PFS [42] (median 20.7 vs. 10.4 months respectively; HR 0.40 (95% confidence interval [CI], 0.20-0.80); *p* = .007), as well as OS (73% vs. 53% at 4 years; HR 0.40 (95% CI, 0.15-1.05); *p* = .055). In DLBCL, several anti-angiogenic drugs (VEGF trap, bevacizumab) have been associated with R-CHOP in first line treatment.

We also demonstrated that VEGF is linked to immune response. In this study, the 57 genes involved in immune response and T-cell activation were decreased in patients with high *VEGF* expression in both ABC-like and GCB-like subtypes of DLBCL, indicating that drugs targeting immune response would be efficient in both subtypes. Various immunotherapies are currently under evaluation in lymphomas [43]. Novel drugs have been reported to be of particular interest in lymphomas such as anti-KIR enhancing NK-cell-mediated cytotoxicity [44], anti-PD1 targeting T-cells infiltrating tumor [45], anti-CD137 targeting immune cells, including NK cells [46].

VEGF isoforms, present numerous differences in matrix-sequestration, transport, and VEGFR/NRP binding, leading to a spectrum of vascular structures, from the stable, thin, and branching vessels of the heavier VEGF_188_ isoform (the murine equivalent of VEGF_189_) to the malformed, oedematous and enlarged network vessels of the most soluble VEGF_120_ isoform (the murine equivalent of VEGF_121_) [47]. The normalization of these pathological vascular structures constitutes the main goal of anti-angiogenic therapies, which may be more successful in tumors that express higher levels of VEGF_121_ leading to normal blood flow patterns and a better cytotoxic drug delivery [48]. It is interesting to note that blockade of VEGFR2 selectively increased blood flow in VEGF_120_-expressing tumors but not in those expressing VEGF_188_ [49, 50].

In conclusion, tumor microenvironment and angiogenesis in DLBCL are differently orchestrated in the ABC-like subtype and in the GCB-like subtype. *VEGF*_*121*_ expression level has a major impact on survival of patients with refractory/relapsed ABC-like DLBCL and is strongly associated with an immune response. This immune response signature is well-conserved in all cancer subtypes and may lead to new therapeutic perspectives. These results need to be confirmed on an independent cohort.

## MATERIALS AND METHODS

The 37 patients studied were a subset of the 477 patients included in the CORAL study [11] which enrolled patients aged 18 to 65 years old presenting a relapsed/refractory CD20+ DLBCL, to compare the efficacy of R-ICE and R-DHAP followed by autologous stem cell transplant (part 1) and to test maintenance with or without rituximab (part 2) [51]. The study was registered under European Union Drug Regulating Authorities Clinical Trials (EudraCT) No.2004-002103-32 and ClinicalTrials.gov NCT 00137995 and was conducted in accordance with Good Clinical Practice rules. All patients gave written informed consent to participate and to provide tissue material for biological studies.

Patients’ characteristics including clinical, histological and GEP scores (to determine COO) [7] and TGS [52]) are summarized in Table 4. The results are part of our previous analysis [10, 12]. Samples used to detect *VEGF* expression level and GEP were the same for each patient.

**Table 4:** Clinical characteristics, Immunohistochemical (IHC) staining results, chromosomal breakpoints analysed by Flurorescent in situ hybridization (FISH) and cell of origin (COO) classification.

Parameters		*n*	%
**Clinical characteristics**			
Sex	Male	29	78
	Female	8	22
Age (years)	Median	50	
	Range	20-63	
PS	0-1	35	95
	2-3	2	5
LDH level	Normal	19	51
	Elevated	18	49
Ann Arbor Stage	I-II	13	35
	III-IV	24	65
Extranodal sites	< 1	24	76
	≥ 2	9	24
Initial response	CR/Cru	27	73
	PR	4	11
	Progression	6	16
Time to relapse	< 12 months	23	62
	≥ 12 months	14	38
Samples	Diagnosis	20	54
	Relapse	17	46
Prior rituximab treatment	Yes	17	46
	No	20	54
Treatment at relapse	R-ICE	19	51
	R-DHAP	18	49
CR at relapse (induction treatment)	Yes	19	51
	No	18	49
**Immunohistochemistry**			
CD10	Positive	13	35
	Negative	24	65
BCL6	Positive	17	47
	Negative	19	53
MUM1/IRF4	Positive	18	49
	Negative	19	51
FOXP1	Positive	20	57
	Negative	15	43
BCL2	Positive	27	73
	Negative	10	27
**Fluorescence *In Situ* Hybridization**			
BCL2/18q21	Positive	12	40
	Negative	18	60
BCL6/3q27	Positive	17	47
	Negative	19	53
c-MYC/8q24	Positive	3	11
	Negative	24	89
**Cell of origin**			
According to Immunohistochemistry	GC	24	65
	Non-GC	13	35
According to Gene Expression Profiling	GC-like DLBCL	19	49
	ABC-like DLBCL	18	51

PS: Performans Status; LDH: Lactate Deshydrogenases; CR: Complete Response.

### VEGF and VEGF receptors evaluation

Expression of 5 angiogenic biomarkers including *VEGF* (isoforms 121, 165, and 189), and their receptors (*VEGFR-1* and *R-2*) was assessed by quantitative qRT-PCR after total RNA extraction and cDNA synthesis from frozen tumor samples, using Perfect-master Mix-probe (Anygenes, Paris, France) on Light-cycler (Roche Diagnostics, Meylan, France) as previously described [53]. The expression levels of the transcripts were normalized to the housekeeping *PPIA* (peptidylpolyl isomerase A) and *TBP* (TATA-box binding protein) gene transcripts. Gene set assays were designed using Primer-Express software (Applied Biosystems, Foster City, CA, USA). Primers and probes sequences are listed in Table 5. Gene expression levels were determined using standard calibration curves prepared form gene-specific PCR products. All PCRs were done in duplicate.

**Table 5:** Specific primer and probe sequences used for real-time RT-qPCR.

VEGF - sense 5’-gAgCTTCCTACAgCACAACAAA-3’ 4 (*)

VEGF 121 - antisense 5’-CTCggCTTgTCACATTTTTC-3’
VEGF 121 - probe 5’-TgCAgACCAAAgAAAgATAgAgCAAgACA 5-8 99

VEGF 206 - antisense 5’-CACCAACgTACACgCTCCAgg-3’
VEGF 206 - probe 5’-AgCAAgACAAgAAAAAAAATCAgTTCgAggAAA-3’ 6a – 6b 146

VEGF 189 - antisense 5’-CCACAgggAACgCTCCAggAC -3’
VEGF 189 - probe 5’-AgCAAgACAAgAAAAAAAATCAgTTCgAggAAA-3’ 6a – 7 144

VEGF 145 - antisense 5’-CTTgTCACATACgCTCCAggAC-3’
VEGF 145 - probe 5’-AAACgAAAgCgCAAgAAATCCCggTA-3’ 6b – 5 145

VEGF 165 - antisense 5’-GCTTTCTCCgCTCTgAgCA-3’
VEGF 165 - probe 5’-AgCAAgACAAgAAAATCCCTgTgggCC-3’ 5 – 7 95

VEGFR-1 - sense 5’-CgACgTgTggTCTTACggAgTA-3’
VEGFR-1 - antisense 5’-CTTCCCTCAggCgACTgC-3’
VEGFR-1 - probe 5’-TgTgggAAATCTTCTCCTTAggTgggTCTC-3’ 24-25 107

VEGFR-2 - sense 5’-TCTCAATgTggTCAACCTTCTAgg-3’
VEGFR-2 - antisense 5’-AAATTTgCAgAATTCCACAATCAC-3’
VEGFR-2 - probe 5’-TgTACCAAgCCAggAgggCCACTC-3’ 19-20 79

B2M - sense 5’CgCTCCgTggCCTTAgC 3’
B2M - antisense 5’ gAgTACgCTggATAgCCTCCA 3’
B2M - probe 5’TgCTCgCgCTACTCTCTCTTTCTggC 3’ 1-2 70

TBP - sense 5’-CACgAACCACggCACTgATT-3’
TBP - antisense 5’-TTTTCTTgCTgCCAgTCTggAC-3’
TBP - probe 5’-TgTgCACAggAgCCAAgAgTgAAgA-3’ 5-6 88

A common *VEGF* forward primer was designed based on the fact that all *VEGF* isoforms share exon 1–5 and various reverse primers or probes were designed to amplify each isoform using its specific sequence mode. VEGF: Vascular Endothelial Growth Factor; VEGFR: Vascular Endothelial Growth Factor Receptor; B2M: Beta2 Microglobulin; TBP: TATA box Binding Protein.

### Statistical analysis

We looked for a prognostic impact of *VEGF* isoforms (121, 165, and 189) and *VEGF* receptors (VEGFR-1 and R-2) expression levels. All survival analyses were performed on an intention-to-treat basis. *VEGF* isoforms, VEGF receptors expression levels and complete remission rates were compared using the chi-squared and Fisher exact tests. PFS was defined as the time from study entry until disease progression or death. OS was defined as the time from the start of treatment until death. Survival functions were estimated using the Kaplan-Meier method and compared with the log-rank test [54]. Differences between the results of comparative tests were considered significant at a 2-sided *p* <0.05. Because the CORAL trial was not stratified by biological data, we controlled for the effects of prognostic factors on outcome due to sampling fluctuations in the treatment groups with a multivariate analysis of survival in a Cox model [55]. All statistical analyses were performed using SAS 9.13 (SAS Institute, Cary, NC, USA) and S-Plus 6.2 (MathSoft, Cambridge, MA, USA) software.

### Gene expression profiling

From the 37 patients, 47 biopsies samples (20 primary biopsies, 17 relapse biopsies and 5 matched cases) were included in the GEP analysis using the Agilent Whole Human Genome microarray (G4112F) (Agilent Technologies, Mississauga, ON, USA). The microarray procedures are previously described [10]. Briefly, total RNA quantity and initial quality were estimated with a NanoDrop^®^ ND-1000 spectrophotometer, and RNA quality was further assessed by electrophoresis with the Agilent 2100 Bioanalyzer (Agilent Technologies). Data have been submitted to the GEO (GSE26812). After raw data normalisation using Lowess method [56], genes with low expression (inferior to median value of the sample) in more than two-third of the samples were rejected. On the 44,000 probes of the microarray, only 14,455 probes went through the filtration step. Genes discriminating for high level (equal or higher than 2) of versus low level (equal or lower than 1) of *VEGF*_*121*_ expression were determined using a t-statistic test at 0.1% risk. Fifty seven genes were found positive and after multi-testing correction for, false-discovery rate was 0.025. Samples were sorted as GCB-like or ABC-like using COO signatures, as described in our previous work [10]. The TGS and the TGS-IPI were applied to the samples considering the expression of *LMO2* and *TNFRSF9* as reported by Alizadeh *et al* [52]. Functional annotations were performed using Gene Ontology (GO) [57] and GoMiner [58]. Significance of over- and underrepresentation of GO terms was computed using Fisher’s exact test. Enrichment of GO terms (frequency of GO term in differential gene list / frequency of GO term in the filtered gene list) was also determined.

### Validation of the signature in public microarray databases

To validate our gene list (*L)*, we mined a large collection of public microarray studies stemming from the public repository GEO. We aimed to validate the coordinated expression trend found in our dataset by systematically analyzing the variation of gene expression among GEO samples. The principle of this discovery process consists in observing the propensity of our gene set to follow the same differential expression between two biological situations. More precisely, we considered a pair of samples (*s*_*i*_, *s*_*j*_) and observed the proportion *p* of positive expression change between samples *s*_*i*_ and *s*_*j*_. Let *s*_*i*_^*k*^ (resp. *s*_*j*_^*k*^) be the expression value of the *k*^th^ *L*-gene observed in sample *s*_*i*_ (resp. *s*_*j*_). The proportion p is defined by the ratio of two numbers: the number of occurrences of positive values of the difference *s*_*i*_^*k*^ - *s*_*j*_^*k*^ ; the number of *L-*genes. A perfect coordinated expression change would correspond to a value *p* = 1. At the opposite, one could expect that independent and identically distributed expression values would result in a value *p* close to 0.5.

Assuming that the sign of *s*_*i*_^*k*^ - *s*_*j*_^*k*^ stems from a fair Bernoulli experiment, we computed the probability to observe the same proportion *p* by chance. We thus obtained a p-value measuring the fitness of a sample pair according to our gene expression signature.

As public datasets are poorly annotated, phenotypes associated to samples are not always clearly determined. Therefore, we followed an unsupervised approach based on the systematic analysis of all pair samples stemming from a GEO dataset. For each GEO study, we followed the same procedure: firstly, samples were preprocessed using rank-based normalization [59]. Then, we computed the p-value for all possible sample pairs. The more significant sample pair was retained as well as its p-value.

This discovery process yielded a collection of GEO series ranked by their sample pair p-values. We selected a set of representative datasets and performed a text mining processing of their annotations (title and summary sections). Using a Fisher test, we explored the most significant terms by comparing the term occurrences between the series related to our selection and the remaining series.

### Functional characterization of the discriminating genes

To identify conserved patterns of co-expression among public microarray datasets, we used a bioinformatics tool called MADCOW [26]. Given a user-specified gene, this online resource extracts strongly co-expressed genes in GEO datasets. More specifically, this tool provides a list of neighbors, a neighbor being a gene having a correlation significantly higher than expected by chance (p-value threshold of 10^-4^). The resulting list comprises a selection of 200 best neighbors (i.e. presenting the highest occurrences in the scanned microarray studies).

From this tool, we identified a set *N* of neighbors stemming from queries based on our gene signature *L*. These results have been modeled as a Boolean matrix *m*(*i*,*j*), were *g*_*i*_ defines a *L*-gene, *g*_*j*_ a gene belonging to *N* and *m*(*i*,*j*) a Boolean indicating if *g*_*j*_ is a neighbor of *g*_*i*_. Matrix *m* can be visualized in terms of a graph in which vertices represent genes and edges describe the neighborhood relationship (*i.e.* an edge is present between two genes if one is the neighbor of the other). As visual exploratory analysis of large graphs is difficult, we simplified the representation using a common technique consisting in drawing a summary of complex graphs [60]. This summary graph is a condensed model that aggregates vertices into a single vertex, each vertex representing a cluster of strongly connected nodes (*i.e.* genes). This summary graph reduces the number of visible elements and then highlights the structure of the initial graph. To construct the summary graph, we performed a hierarchical clustering starting with nodes corresponding to single genes. Then, pairs of connected nodes were iteratively joined to form dense nodes equivalent to clusters. In our agglomerative procedure, two nodes were joined if and only if the resulting cluster remained a fully connected component (this required that all the possible connections inside a cluster were met: all the *L*-genes must be interconnected and every neighbor must be connected with all the *L*-genes). An edge between two clusters was drawn if their inter-cluster connectivity was greater or equal to a predefined threshold. Inter-cluster connectivity was defined as the proportion of edges (*g*_*1*_, *g*_*2*_) between clusters *c*_*1*_ and *c*_*2*_, where *g*_*1*_ (resp. *g*_*2*_) belongs to *c*_*1*_ (resp. *c*_*2*_).

## Abbreviations

ABC: Activated B-Cell like
AIF1: Allograft Inflammatory Factor 1
BCL11B: B-Cell leukemia/lymphoma 11B
BCR: B-Cell Receptor
CNV: Copy Number Variation
COO: Cell Of Origin
CORAL: Collaborative study on Relapsed Aggressive Lymphoma
CR: Complete Response
DLBCL: Diffuse Large B-cell Lymphoma
FDR: False Discovery Rate
FISH: Fluorescence In Situ Hybridization
GCB: Germinal Center B-cell like
GEO: Gene Expression Omnibus
GEP: Gene-Expression Profiling
GO: Gene Ontology
GSE: GEO Series
IPI: International Prognosis Index
LDH: Lactate DesHydrogenase
MHC: Major Histocompatibility Complex
OS: Overall Survival
PFS: Progression-Free Survival
PI3K: Phosphoinositide 3-Kinase
PPIA: Peptidylpolyl isomerase A
PS: Performance Status
R-CHOP: Rituximab, Cyclophosphamide, Adriamycin, Vincristine and Prednisone
R-DHAP: Rituximab, Dexamethasone, Cytarabine, Cisplatine
R-ICE: Rituximab, Ifosfamide, Carboplatine, Etoposide
SD: Standard Deviation
SOCS1: Suppressor Of Cytokine Signaling 1
SSI: STAT-induced STAT Inhibitor
TBP: TATA-box binding protein
TGS: Two-Gene Score
VEGF: Vascular Endothelial Growth Factor
VEGFR: Vascular Endothelial Growth Factor Receptor
